# Pattern of Presentation and Outcome of Short-term Treatment for Idiopathic Clubfoot / CTEV with Ponseti Method

**DOI:** 10.5704/MOJ.1611.009

**Published:** 2016-11

**Authors:** R Gunalan, A Mazelan, YPB Lee, A Saw

**Affiliations:** Department of Orthopaedic Surgery, University Malaya Medical Centre, Petaling Jaya, Malaysia

**Keywords:** Foot, relapsed, resistant, foot deformity, congenital, Ponseti method

## Abstract

**Introduction:** Congenital Talipes Equinovarus (CTEV) is a common congenital foot deformity that is associated with long term disability. Treatment with Ponseti method has been successful especially for children who present early. We conducted this study to investigate the age of presentation of children and report the early outcome.

**Materials:** This is a retrospective study from a single institution. We included 31 patients with 45 idiopathic clubfeet and investigated problems and success rate at the end of serial casting.

**Results:** Mean age at presentation was 4.9 months. The mean number of casting was 6 and mean duration of casting was 2.7 months. The initial success rate of 91.1%, with four feet (8.8%) diagnosed as resistant clubfoot and eventually required soft tissue surgery. With mean follow up of 14.1 months, four other feet (8.8%) developed relapse but were treated with repeat Ponseti method.

**Conclusion:** Many CTEV patients present late for treatment. However, the Ponseti method remained effective with high initial success rate of 91.1%. Relapsed CTEV can still be treated successfully with repeat casting using the Ponseti method.

## Introduction

Congenital talipes equinovarus (CTEV) also known as clubfoot, is a relatively common congenital foot deformity with an incidence of 1 per 1000 live births^1^. The deformity is characterized by hindfoot equinus, midfoot varus, and forefoot supination/adduction. If left untreated, the deformity will persist and gradually become rigid due to secondary changes in the tarsal bones and joints^2^. Persistent weight bearing over the lateral border or dorsolateral aspect of the foot will result in callus formation, pain on walking, and inability to wear standard shoes. These may eventually predispose to skin break down, substantial reduction in level of ambulation and limitation in employment opportunities^1^.

Extensive soft tissue surgery had been considered as the optimal treatment for idiopathic clubfoot^2^. In the late 90s, non-operative treatment gradually became popular following publication of long term treatment outcome by Ponseti and his team^3,4,5^. Increasing evidence has shown that extensive surgical release is associated with stiff and arthritic foot during adulthood^2^. With the promotion by orthopaedic surgeons in various institutions, the Ponseti method has gained popularity all over the world including in many developing and underdeveloped countries^6,7,8^.

Diagnosis of clubfoot is generally clinical since the deformity is obvious at birth. In less developed countries, late presentation is common due to lack of awareness, availability of treatment or delay in referral. Some parents may refuse treatment and decide to seek traditional methods of treatment. In 1990, Boo reported an incidence of 4.5 per 1000 live births based on data from Maternity Hospital Kuala Lumpur, Malaysia^9^. More recently, Rasit reported treatment outcome of 30 children with CTEV in East Malaysia where 18 of them required surgery due to failure of non-operative treatment^10^. The most recent study in this region was by Awang *et al* in 2014^11^. We conducted this study to look at the pattern of presentation and short-term treatment outcome with the Ponseti method.

## Materials and Methods

This is a retrospective cohort study on children presenting with CTEV to our institution. We adopted the Ponseti method of treatment after our doctors and plaster technicians attended a Ponseti training workshop organised by the Paediatric Orthopaedic Society of North America (POSNA) in 2004. After obtaining ethical committee approval from the institution (ID number 201512-1927), we retrieved medical folders of all the patients treated in the weekly clubfoot clinic. Number of patients was based on those attending the clubfoot clinic from 1st January 2013 to 31st Dec 2014 (period of 2 years). Patients with underlying neuromuscular conditions, syndromes, and deformities related to trauma, tumour and infection were excluded from the study. Those with flexible clubfeet that did not require any manipulation or casting were also excluded.

All the patients with CTEV were treated with weekly foot manipulation and change of plaster casts according to the principles and techniques described by Ponseti^4^. Figure 1a shows a patient with clubfoot deformity before any serial casting was attempted. Percutaneous Achilles tenotomy were performed on selected feet to correct equinus deformities. Achilles tenotomy was done when the foot was passively able to achieve at least sixty degrees of external rotation in relation to the long axis of the tibia. This enabled the foot to be held in at least fifteen degrees of dorsiflexion in the final cast. In our institution, tenotomy procedures were most commonly performed under general anaesthesia. The final cast was applied for three consecutive weeks, after which the patients were required to use an abduction orthosis. Figure 1b shows a patient, six weeks after serial casting treatment was done.

**Fig. 1a:**
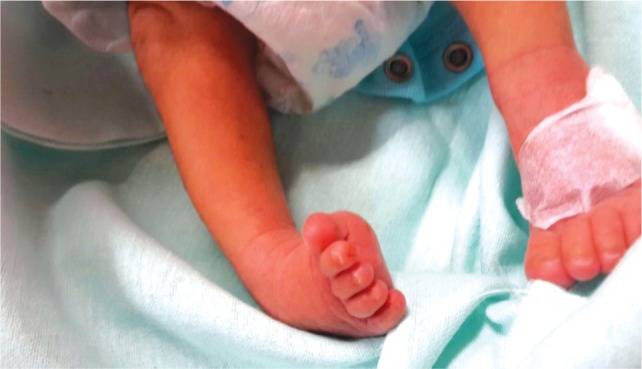
Before casting applied.

**Fig. 1b:**
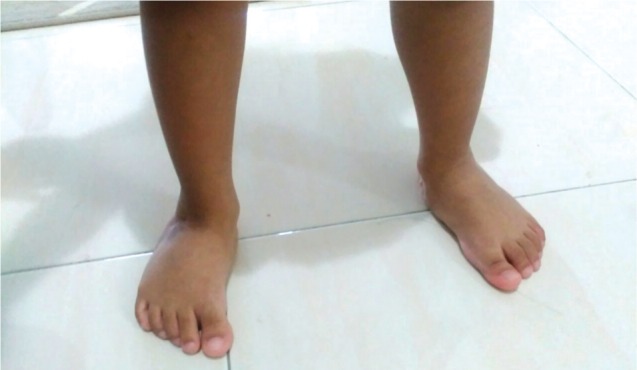
After completing casting. Patient able to reach plantigrade position with flexible residual adduction of the forefoot.

Parents were advised to use full time shoe abduction orthosis until the child started pulling up to stand. They were advised to use the abduction orthosis for twenty-three hours a day. One of the reasons for using the orthosis till this age was that many children presented to us several months after birth, and by the time they completed casting it would be not too long before they started to stand and eventually walk. After that, the child would only wear the abduction orthosis for about six hours at night. We recorded relevant information on each patient that included demographic data, age on presentation, casting procedure and clinical outcome on follow up. Deformities that were fully corrected but subsequently recurred were considered as relapsed clubfeet. A fully corrected deformity was defined as those feet that we were able to achieve a corrected midfoot position with the absence or minimal medial crease, minimal curved lateral border and non-palpable lateral head of talus. The hindfoot was considered fully corrected if there was an absence of the posterior crease and the foot could reach at least a plantigrade position. Those that were not fully corrected after 10 changes of casts, or required surgical intervention (excluding percutaneous tenotomy) were considered as resistant clubfeet.

All the information was recorded in Microsoft Access database file. Selected data sets were subsequently transferred to Microsoft Excel program for further analys.

## Result

There were 31 children with 45 clubfeet treated in the institution during the study period. There were more boys (23) compared to girls (8), with a ratio of 2.8:1, with 14 (45%) bilateral deformities, and 17 (55%) unilateral deformities. When unilateral, the right feet were more commonly affected (12, 71%) than the left feet (5, 29%).

**Fig. 2:**
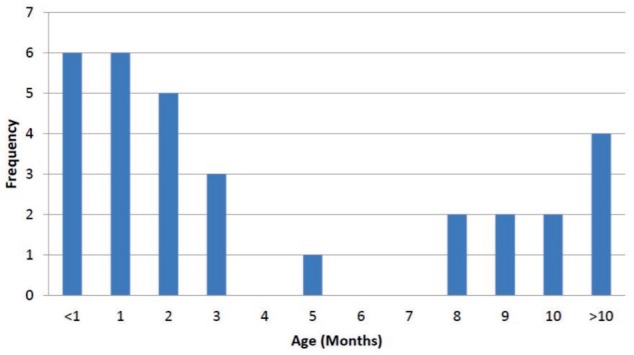
Ages at which the patients presented to our clinic with the diagnosis of CTEV. This indicates the age at which the treatment with serial casting was commenced.

**Fig. 3:**
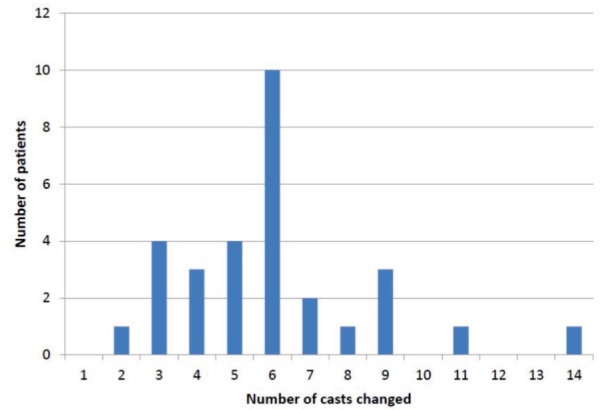
Number of cast change required for our patients. This indicates the response of the patient to this method of treatment.

**Fig. 4:**
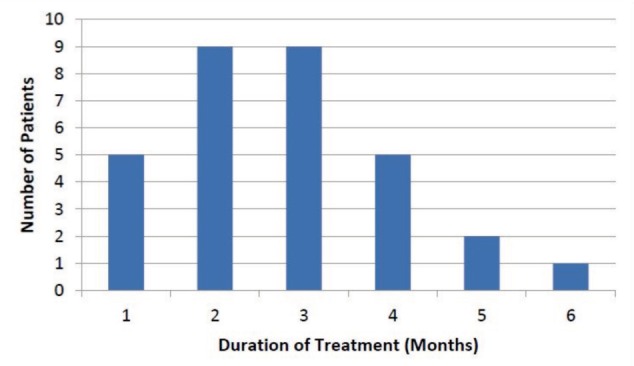
Duration (in months) patients treated for CTEV in our clinic. This indicates the period of time this method of treatment was provided for these patients.

The mean age of patients at presentation was 4.9 months (Figure 2). There were only six patients who presented within four weeks after birth, and one patient was referred at the age of 21 months for treatment. This patient presented at a late stage, as he had been treated in a different medical facility prior to being referred to our centre. The mean number of cast change for all the feet was six ranging from three to 14 changes (Figure 3). The mean duration of casting was 2.7 months (range: 1 – 6 months) (Figure 4). Of the 31 patients, 18 (58%) required percutaneous Achilles tenotomy.

Out of our 31 patients with 45 club feet, we were not able to correct the deformity of three patients (9.6%) with 4 club feet (8.8%) and they were diagnosed to have resistant clubfoot (Figure 2). They subsequently required soft tissue surgery for the correction of their deformities. With 41 of the 45 feet responding to treatment, our initial success rate was 91.1% (Figure 1b). The mean period of follow up was 14.1 months, ranging from four to 30 months. Of the remaining 41 patients who were successfully treated, four feet subsequently developed unilateral relapse, giving rise to relapse rate of 9.8%. The initial presentations of these four cases did not vary significantly from the others, in terms of the severity of their deformity or the resistance to passive correction. However, as the serial casting was applied, it was noted that the progress in these four patients was slower than the other patients in this report. There was no underlying pathological differences between the cases. However, the compliance to abduction brace wear was poor among these four patients. All of them were successfully treated with repeated serial casting and a repeat percutaneous heel cord tenotomy. One patient required a medial soft tissue release in combination with a tendo-Achilles lengthening procedure.

## Discussions

In our study, the number of boys was close to three times the number of girls, with a ratio of 2.8:1. This is contrary to local studies conducted by Boo^9^ and Rasit^10^ that showed equal number of patients between the two genders. However, studies from Sweden^12^ and West Australia^13^ reported male predominance with the ratio of 2.4 and 2.0 to 1 respectively.

Slightly more than half of our patients had unilateral clubfoot (55%), and this is consistent with studies by Rasit^10^ and Wallander^12^ who reported 67% and 54% of unilateral cases respectively. We had more right sided (71%) unilateral clubfoot. In the literature, Byron^14^ and Wallander^12^ reported that right unilateral clubfoot was more common, while other studies by Boo^9^ and Rasit^10^ reported that the left side was more commonly affected.

The initial success rate of treatment by serial manipulation and casting reported by Ponseti and Smoley^15^ in 1963 was 80%. From the 45 clubfeet that we treated, we were not able to achieve full correction in four feet (8.8%) even after tenotomy, giving rise to an initial success rate of 91.1%. This is a relatively good result considering that the mean age at presentation of our patients was 4.9 months, and that ten out of 31 patients (32.3%) came for treatment after six months of age. Lehman *et al*^16^ reported 92% success rate in treatment of younger children less than 7 months old. A recent study by Verma *et al*^17^ reported an initial successful rate of 89% in older children aged between one to three years.

In our study, we observed a relapse rate of 9.8%. This is consistent in comparison to the more recent experience from Morcuende^18^ who reported a relapse rate of about 10%. Higher relapse rates had been reported by Panjavi^6^ and Changulani^19^ at 18.6% and 32% respectively. One of the main reasons for developing relapse was lack of compliance with using abduction shoe orthosis. Some parents admitted that they had problem with regular wearing of abduction shoe splint for their children, and occasionally, the supplier was not able to provide the proper size splints in time. Nogueira *et al*^20^ had highlighted similar problems with regular supply of orthosis and parental compliance in South America. We are trying to solve the issue by creating awareness of this condition and its proper treatment through public health education.

Although four of the 45 (8.8%) feet were diagnosed as resistant clubfeet and eventually required extensive soft tissue surgery, subsequent relapse in four feet were successfully treated with repeat serial manipulation and casting. Although we would expect more cases of relapse with longer follow up, they may only require similar treatment, or more limited type of surgical procedures. Ponseti and Smoley^15^ reported that open surgery was avoided in 89% of cases. Changulani on the other hand reported that 81% of their cases could be corrected without the need for soft-tissue release^19^.

The main limitation of this study is the small number of patients and short follow up. However, the study was able to show that many patients in this country presented late and more effort should be organised to improve awareness on the need for early treatment. Further studies with longer follow up that include compliance with the use of orthotic shoes would help us to better understand the long term outcome of treatment in this region.

## Conclusion

With the initial success rate of 91.1%, our study showed that the Ponseti method of treatment was effective in the treatment of CTEV even for those who presented late (mean of 4.9 months after birth). However, we were not able to eliminate the need of soft tissue surgery in four of the resistant clubfeet (8.8%). During the short follow up, four relapsed clubfeet were diagnosed but all of them responded with repeated treatment using the Ponseti method.
